# Surveillance Screening in Li-Fraumeni Syndrome: Raising Awareness of False Positives

**DOI:** 10.7759/cureus.2527

**Published:** 2018-04-24

**Authors:** Prerna Kumar, Ryan M Gill, Andrew Phelps, Asmin Tulpule, Katherine Matthay, Theodore Nicolaides

**Affiliations:** 1 Pediatrics, University of California, San Francisco, San Francisco, USA; 2 Pathology, University of California, San Francisco, San Francisco, USA; 3 Radiology and Biomedical Imaging, University of California, San Francisco Benioff Children's Hospital, San Francisco, USA; 4 Pediatric Hematology/Oncology, University of California, San Francisco, San Francisco, USA; 5 Pediatrics, NYU Langone Medical Center, New York, NEW YORK, USA

**Keywords:** cancer prevention, surveillance screening, sick kids protocol, false positives, incidental findings, li-fraumeni syndrome

## Abstract

Li-Fraumeni syndrome (LFS) is a rare cancer predisposition syndrome inherited in an autosomal dominant fashion that involves a germline mutation of tumor protein 53 (TP53). With the advent of more accessible and accurate genetic testing methods, along with more widespread knowledge of LFS, asymptomatic carriers can now be more easily identified. No general surveillance protocols were previously recommended other than routine physical exams and breast and colon cancer screening at younger ages, primarily due to questions involving efficacy, cost, and clinical benefits. With more data now available to support the implementation of a surveillance protocol for cancer predisposition syndromes such as LFS, preventative screening has become a national standard of care. However, as surveillance becomes more integrated into patient care, the benefits and risks must be further evaluated. We briefly describe our institutional experience with surveillance screening in LFS and describe two patients in depth where surveillance imaging brought to light false positives that led to increased utilization of resources and concern for new malignancy. Though the benefits of surveillance are clear, it is important to understand the potential for false positives involved with instituting this practice. Continued research of this topic is thus warranted, perhaps with larger prospective studies, to better capture the survival benefits of patients undergoing surveillance screening and more comprehensively understand the incidence of false positives.

## Introduction and background

Li-Fraumeni syndrome (LFS) is a hereditary cancer syndrome that increases the likelihood of developing cancer over one’s lifetime. Individuals affected with LFS have a 50% chance of developing cancer by the age of 30 years and a 90% chance of developing cancer by the age of 60 years [1]. Bougeard et al. showed that in French patients with LFS, the incidence of a pediatric malignancy was 22% by the age of five years and 41% by the age of 18 years [2]. This incidence represents the risk of malignancy for families with strong cancer predisposition syndromes and likely tumor protein 53 (TP53) null mutations; it is possible, however, that as more mutations involving slightly more functional TP53 alleles are identified, the incidence of malignancy in certain subsets of the Li-Fraumeni population may be different. The core cancers associated with LFS are sarcomas (including soft tissue sarcomas and osteosarcoma), pre-menopausal breast cancer, brain tumors, adrenocortical carcinoma (ACC), and leukemias. In the past, the diagnosis of LFS was made once enough clinical criteria were met, including the number and ages of affected family members and the types of specific cancers diagnosed in the family. However, since a germline pathogenic TP53 mutation is the only gene known to be implicated in LFS, targeted gene sequencing can now quickly identify both affected and unaffected carriers. Malkin et al. reported that 95% of pathogenic germline TP53 variants can be detected via sequencing the entire coding region (exons two through eleven) and that 80% of patients with LFS should have a detectable variant by sequencing [3]. It is important to note, however, that targeted gene sequencing will not identify TP53 rearrangements, such as that seen in osteosarcoma; thus, more comprehensive approaches such as whole exome sequencing or ribonucleic acid (RNA) sequencing may need to be employed in those settings. If a patient does not have a concerning family history, but presents with a suspicious personal medical history, LFS should still be considered, particularly since 7-20% of LFS is due to *de novo* mutations [4]. Based on the literature, it has been demonstrated that choroid plexus carcinoma (CPC) may be an indicator of an existing TP53 mutation irrespective of family history [5, 6].

Rapid advances in genetic testing technology have made the diagnosis of inheritable conditions much easier; however, the question of how best to proceed with the resulting genetic information has sparked controversy and conversation. Historically, with cancer predisposition syndromes like LFS, genetic testing was often not pursued because it did not significantly change clinical management for those families who were clinically at increased risk. Patients were told to continue routine physical exams with their primary care provider in addition to having earlier breast exams and mammograms for breast cancer screening and earlier colonoscopies for colon cancer screening. Whole body magnetic resonance imaging (MRI) was not part of the surveillance strategy given concerns for accurate interpretation with subsequent false positives as well as the balance of costs and benefits. In addition, the recommendations differed greatly for adult versus pediatric patients, particularly given the unique challenges of surveillance in the pediatric population - specifically more blood draws (which can be considered relatively invasive for some families) and the need for sedation or anesthesia in order to obtain high quality images. Nonetheless, several studies have now demonstrated the role of whole body surveillance in LFS with the goal to detect cancer earlier, prevent advanced stage disease at diagnosis, and improve overall survival. Though the described results are impressive, it is important to note that the overall number of patients studied is small. Thus, though whole body surveillance is a newly emerging recommendation in LFS, it continues to warrant further study.

## Review

Surveillance screening in Li-Fraumeni syndrome

Villani et al. demonstrated the feasibility of a surveillance protocol in asymptomatic germline TP53 mutation carriers and showed that in a small group of eight families, comprehensive screening resulted in early detection of tumors and improved overall survival [7]. In their prospective observational study, they identified 33 TP53 mutation carriers, 18 of whom chose to undergo surveillance. Seven patients were found to have asymptomatic tumors and underwent appropriate treatment, and all seven patients were alive after 24 months (median follow-up time). Ten patients developed tumors in the non-surveillance group – all higher grade and stage than in the surveillance group – and only two patients were alive at the end of follow-up [7]. This prompted a follow-up study that built upon and confirmed the initial results of the prospective pilot (from 2004-2010, n=33) and involved longer follow-up and more patients (from 2004-2015, n=89) [8]. Villani et al. demonstrated again, that with a comprehensive clinical screening protocol including physical exams, labs, and frequent imaging with a combination of whole body MRI, brain MRI, breast MRI, mammography, abdominal and pelvic ultrasound (U/S), and colonoscopy, individuals with TP53 mutations had earlier detection of asymptomatic tumors and improved long-term survival compared to their unscreened counterparts (five year overall survival 88.8% versus 59.6%) [8]. Of note, the authors disclosed two false negatives (cancer diagnosed between surveillance assessments) and two false positives (lesions that were biopsied and subsequently found to be non-neoplastic by pathology).

Based on the above experience of the Hospital for Sick Children in Toronto, Canada, the “Sick Kids Protocol” or the “Toronto Protocol” is widely accepted as a surveillance strategy for children with TP53 mutations, grouped by cancer type, as listed below [7, 8]. Of note, components that have been modified from the original Sick Kids Protocol or not recommended by the American Association of Cancer Research (AACR) are included in brackets for easy reference [9]. Lastly, it is important to emphasize the role of annual comprehensive physical exams to evaluate for hypertension, growth, cushingoid appearance, signs of virilization, and/or changes in the neurologic exam.

Current recommendations for surveillance screening

*Adrenocortical Carcinoma* [until age 18 years*]

U/S of abdomen and pelvis every three to four months

Complete urinalysis every three to four months

Blood tests every three to four months: beta-human chorionic gonadotropin (b-HCG), alpha-fetoprotein (AFP), 17-hydroxyprogesterone (17-OH-progesterone), testosterone, dehydroepiandrosterone sulfate (DHEAS), androstenedione [to be ordered if U/S insufficient]

*Due to very low incidence of ACC in adults and highest risk in very young children

Brain Tumor

Annual brain MRI [with contrast initially and if normal, can be completed without contrast to minimize the accumulation of contrast in the basal ganglia from repeated MRIs]

Soft Tissue and Bone Sarcoma

Annual rapid total body MRI*

*At our institution, whole body MRI typically takes 30-90 minutes, depending on the patient and the scanner. Our institution’s specific protocol is further outlined in Table 1. The technical and practical aspects of acquiring and interpreting whole body MRI studies, as well as the issues regarding sedation and the use of gadolinium in the pediatric population, are important given the widespread adoption of whole body MRI in surveillance. Though this is out of the scope of this review, these issues are very nicely addressed in further detail by Greer et al. and Goo et al. [10, 11].

**Table 1:** Institution Specific Whole Body MRI Protocol

Timing of image acquisition	Sequences obtained
Before gadolinium	Whole body coronal short tau inversion recovery (STIR)
Before and after gadolinium	Whole body coronal three-dimensional spoiled gradient (SPGR) with Dixon method of fat suppression
Optional sequences	Targeted axial T2 fast spin echo (FSE) with fat saturation and targeted axial diffusion weighted imaging (DWI)

Leukemia or Lymphoma

Blood tests every three to four months: complete blood count (CBC), erythrocyte sedimentation rate (ESR), lactate dehydrogenase (LDH) [to be ordered if concerning physical exam or history]

The surveillance strategy now recommended for adults includes everything described above with the addition of the following:

Breast Cancer*

Routine self-exams monthly starting at age 18 years supported by clinical breast exams twice yearly starting at age 20-25 years (or five to 10 years before the earliest known breast cancer in the family)

Annual breast MRI alternating with whole body MRI [one scan every six months] beginning at age 20-25 years (or five to 10 years before the earliest known breast cancer in the family) [mammography or U/S has been omitted]

Consideration of risk-reducing bilateral mastectomy

*Males with LFS are not known to have an increased risk of breast cancer

Colon Cancer and GI Tract Malignancies

Endoscopy and colonoscopy every two to five years beginning at age 25 years or 10 years before the earliest known colon cancer in the family

Melanoma

Annual skin exam by dermatology beginning at age 18 years

Institutional experience with surveillance screening

At our institution, we began following this protocol in the last several years and are currently managing seven LFS patients with surveillance screening (Table 2). We will briefly review these seven patients and present our experience with surveillance screening in LFS.

**Table 2:** Institutional Experience with Surveillance Screening

Patient	First diagnosis, age at diagnosis	Second diagnosis, age at diagnosis	Surveillance screening findings	Age at last follow-up/comments
1	CPC, three years old		Iron overload discovered on surveillance requiring phlebotomy	Five years old
2	CPC, five months old		Focal nodular hyperplasia	Seven years old
3	Neuroblastoma, two months old	Grade II astrocytoma, six years old		12 years old
4	Left orbital rhabdomyosarcoma, 13 months old	Left temporal osteosarcoma (in the radiation field), 11 years old; local recurrence of osteosarcoma, 13 years old, 15 years old	Benign thyroid nodule	15 years old
5	N/A	N/A		11 years old, sibling of affected patient
6	Right distal femoral osteosarcoma, five years old			Nine years old
7	N/A	N/A		Eight years old, sibling of affected patient

Patient 3 was diagnosed with intermediate risk neuroblastoma at age two months with a left adrenal primary and liver metastases and achieved complete response with eight cycles of chemotherapy alone. A very strong and concerning maternal family history of cancer prompted genetic testing for the patient and his mother and revealed a TP53 mutation (TP53p.E286K). Surveillance screening was initiated that detected a low grade glioma at age six years, which was resected and confirmed to be infiltrative grade II astrocytoma.

Patient 4 was diagnosed with left orbital rhabdomyosarcoma at age 13 months and treated with chemotherapy and external beam radiation. A left temporal osteosarcoma (in the radiation field) was then diagnosed at age 11 years treated with chemotherapy and surgical resection. Because of a concerning family history, the diagnosis of LFS was made at the end of therapy. The patient and her mother have an exon five tandem duplication of the TP53 gene, which, of note, was not identified by targeted sequencing and required special testing. Surveillance screening was initiated, which identified a local recurrence of osteosarcoma two years after completion of therapy. This was treated with surgical resection, gamma knife radiation, and chemotherapy with ifosfamide alone (no etoposide given the known increased risk for secondary malignancy). Surveillance screening also demonstrated a thyroid nodule on whole body MRI that required biopsy and was ultimately benign. This patient has a sibling, patient 5, with the same germline mutation but no cancer diagnoses, who is also followed by surveillance screening and has had no identified abnormalities.

Patient 6 was diagnosed with osteosarcoma of the right distal femur at age five years and treated with chemotherapy and surgery. A concerning family history on both maternal and paternal sides prompted genetic testing, which demonstrated a TP53 mutation (TP53p.C141W) that was found in the patient and his father. The patient continued surveillance screening with no further findings thus far at age nine years. This patient has a sibling, patient 7, with the same germline mutation but no cancer diagnoses, who is also followed by surveillance screening and has had no identified abnormalities.

The remaining two patients form the basis of this review and are described in detail below.

Case Report: Patient 1

Patient 1 is a five-year-old male who presented in status epilepticus and was found to have CPC with no spine metastases at age three years. He was treated with gross total resection, chemotherapy per Children's Oncology Group (COG) study ACNS-0333 (three cycles of induction with vincristine, methotrexate, etoposide, cyclophosphamide, and cisplatin) and three tandem autologous transplants (including consolidation with carboplatin and thiotepa). He was found to have a *de novo* germline TP53 mutation (TP53p.M246V), as his parents and sibling were all negative. He has been off therapy since 2015 and continues to receive every three month U/S with urine analysis and labs along with annual whole body and brain MRI. Therapy complications included an etoposide allergy and grade three chemotherapy associated hearing loss, which required dose reduction. He experienced a second seizure off therapy (the first was in the setting of his diagnosis) but is otherwise doing well. The patient also has a diagnosis of Lynch syndrome, known as hereditary nonpolyposis colorectal cancer (HNPCC), due to the presence of one mutated allele of MutS Homolog 6 (MSH6) (MSH6p.T716fs) on his genetic testing results. This mutation was inherited from his father who underwent testing at the time of the patient’s diagnosis.

Early routine surveillance screening revealed an elevated AFP in laboratory screening but no evidence of a mass on whole body MRI screening. Serum ferritin was checked as this can be associated with an elevated AFP. The ferritin was abnormally elevated to 1341 micrograms per liter (ug/L) (normal reference range 22-322 ug/L), which prompted a workup for iron overload, given that the patient was otherwise well with no concern for persistent inflammation or malignancy. To evaluate for hepatic and cardiac iron overload, T2* imaging was completed and revealed diffuse iron deposition of the liver associated with mild transaminitis. The patient was overall completely asymptomatic with a normal physical exam. Genetic testing for hemochromatosis, given the unclear etiology of the iron overload in this setting, was negative for the most common mutations. Because of the risk for hepatic cirrhosis due to hepatic iron overload, treatment was recommended. Given the risk of renal toxicity, ototoxicity, and bone marrow toxicity associated with chelation – all of which the patient had already experienced secondary to his prior CPC therapy – phlebotomy was initiated every two weeks. The degree of iron overload did appear out of proportion to what would be expected for an autologous stem cell transplant recipient who had required only 19 red blood cell transfusions. The patient is doing well, aside from emotional distress over this therapy plan, and his ferritin has normalized nicely, as has his AFP.

Case Report: Patient 2

Patient 2 is a six-year-old female with a history of CPC diagnosed at age five months with positive cerebrospinal fluid (CSF) but no frank metastases. She was treated with gross total resection, chemotherapy per COG study ACNS-0334 (including induction with vincristine, methotrexate, etoposide, cyclophosphamide, and cisplatin), one autologous transplant (including consolidation with carboplatin and thiotepa), and intrathecal liposomal cytarabine (Ara-C). She was found to have a germline TP53 mutation (TP53p.A248G). Her parents declined testing and refused testing of other family members. The patient has been off therapy since 2011 and continues surveillance screening. Therapy complications included placement of a shunt for hydrocephalus, chemotherapy-associated hearing loss, and developmental delay.

**Figure 1:**
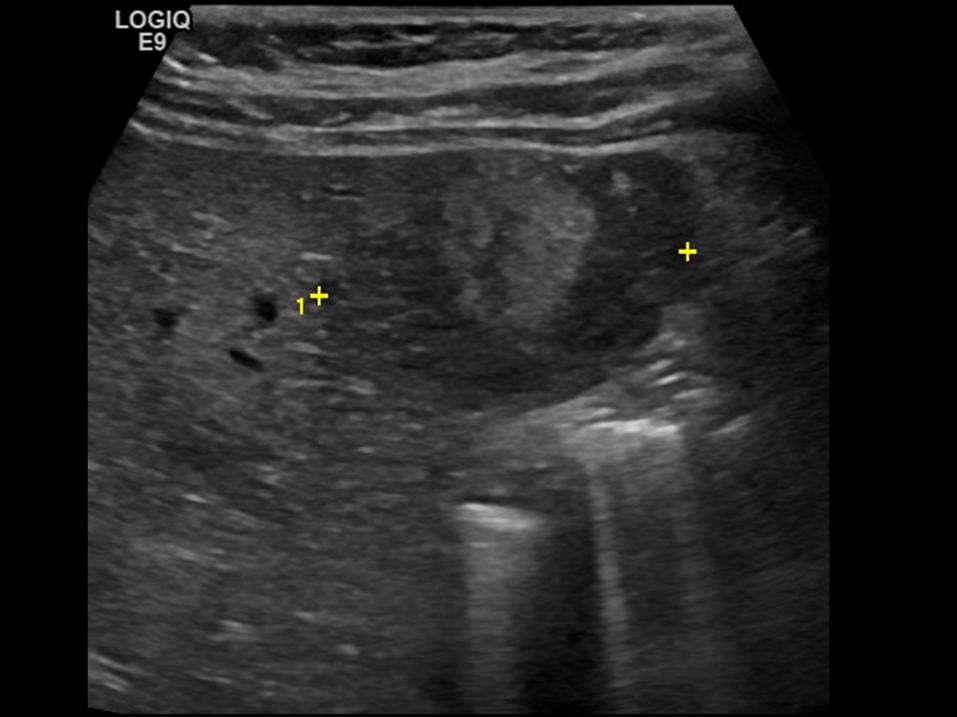
Transverse Ultrasound Image of the Liver Transverse ultrasound image of the liver demonstrating a 3 centimeter heterogeneous lesion in the left inferior liver edge (indicated by measurement calipers). This particular lesion retrospectively corresponded with the area of subtle T2 hyperintensity on initial whole body MRI and is also the lesion that would ultimately get resected (with pathology confirming focal nodular hyperplasia). Several additional similar-appearing lesions were also detected on this ultrasound study (not included in this image).

**Figure 2:**
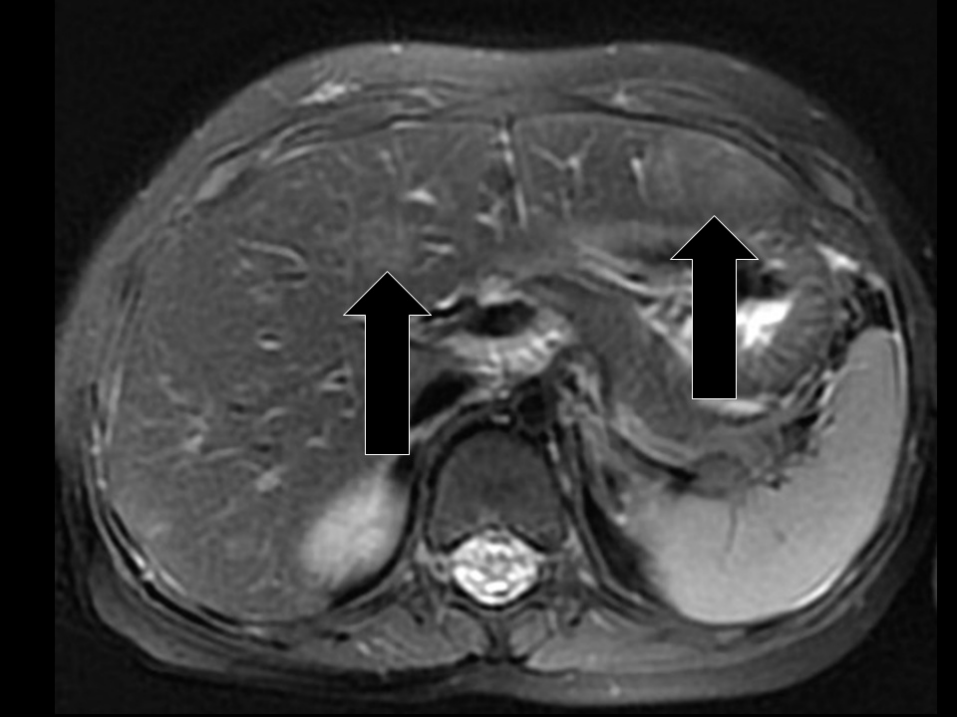
Axial T2-weighted Sequence Obtained as Part of a Dedicated Liver MRI Study to Further Evaluate the Lesions Seen by Ultrasound On this MRI study, a total of seven liver lesions were detected, three of which were not detected by ultrasound and two of which are shown on this image (indicated by arrows). The left liver edge lesion corresponded with the lesion demonstrated in Figure 1.

**Figure 3:**
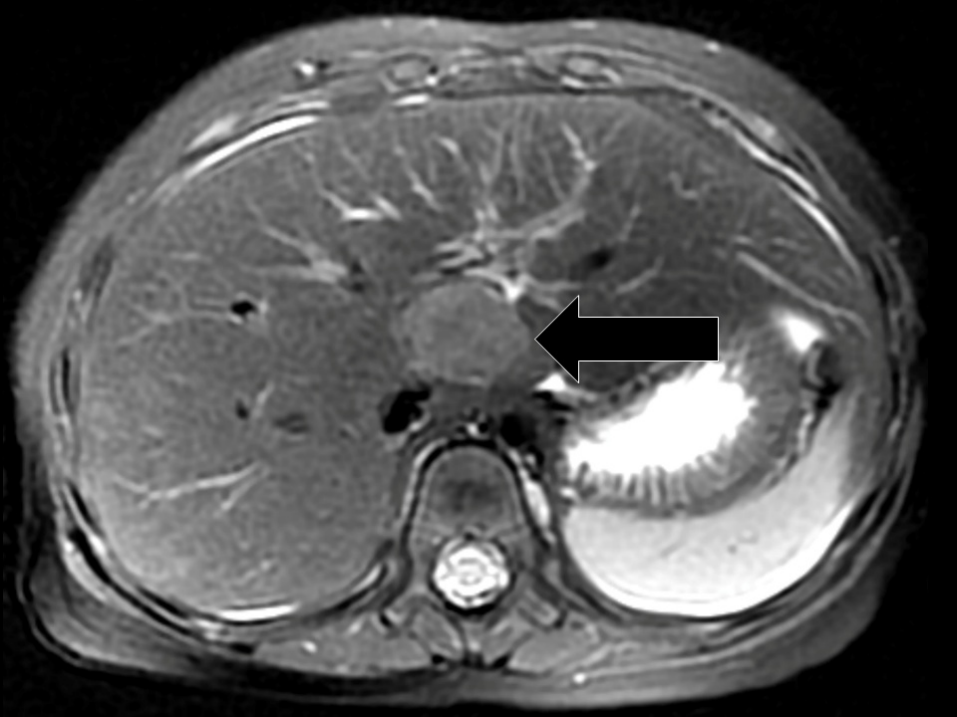
Axial T2-weighted Sequence Axial T2-weighted sequence showing a more centrally located liver lesion (indicated by arrow). While this particular lesion was the most conspicuous by MRI, it was inconspicuous by ultrasound.

**Figure 4:**
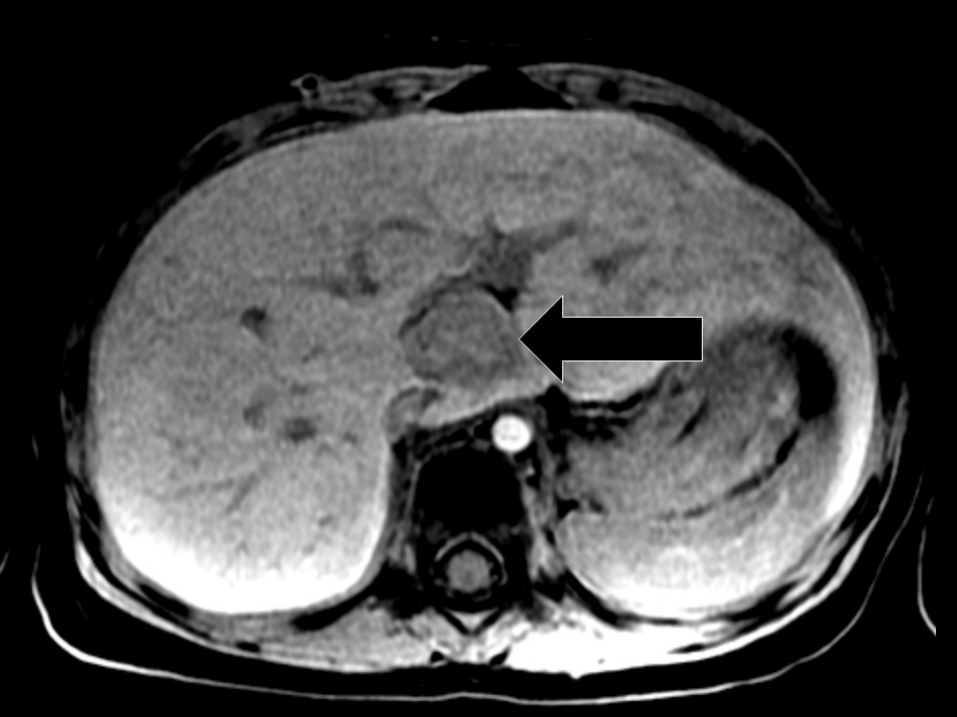
Axial T1-weighted Sequence Obtained after Intravenous Injection of Eovist Axial T1-weighted sequence obtained 20 minutes after intravenous injection of the hepatobiliary imaging agent Eovist (gadoxetate disodium). There was variable Eovist uptake in the liver lesions seen on T2-weighted imaging. In this centrally-located lesion (indicated by arrow), there is poor Eovist uptake compared with background liver parenchyma. Functional hepatocytes are required for Eovist uptake, and FNH typically contains functional hepatocytes; therefore, poor Eovist uptake is concerning for a non-hepatocyte-containing neoplasm. In this patient, this imaging expectation led to a false positive result. FNH - focal nodular hyperplasia.

**Figure 5:**
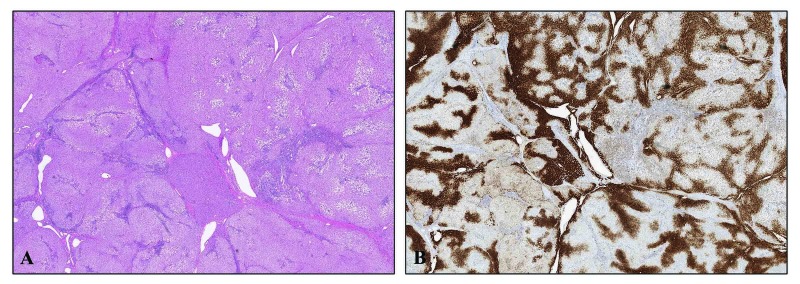
Focal Nodular Hyperplasia (FNH) (A) Hematoxylin and Eosin stained section of FNH demonstrating characteristic fibrous tissue and patchy fatty change in a portion of the resected tumor, 40x magnification. (B) Glutamine synthetase immunohistochemical stained section, depicting the same region of the tumor as presented in Part A, demonstrating a map-like staining pattern characteristic of FNH, 40x magnification.

Routine whole body MRI screening revealed multiple liver lesions of unclear etiology concerning for malignancy. This prompted an extensive workup including repeat imaging with U/S and MRI over the course of a few months to assess for change in size or heterogeneity. Dedicated liver U/S demonstrated a 3 centimeter (cm) heterogeneous lesion in the left inferior liver edge (Figure 1) which corresponded retrospectively with an area of subtle T2 hyperintensity on whole body MRI. Dedicated liver MRI study detected seven lesions, three of which were not detected by U/S (Figures 2, 3). Overall, imaging confirmed the presence of multiple enhancing hypointense lesions with reduced diffusion and relatively unchanged size. The most concerning diagnosis on the differential was certainly a malignant neoplasm, most likely metastases, which could not be excluded by imaging alone; however, the differential diagnosis of non-malignant etiologies primarily consisted of focal nodular hyperplasia (FNH), which is the second most common benign liver tumor (after cavernous hemangioma). FNH is a nodular hepatic lesion that can be single or multiple and has been associated with vascular malformations as well as brain neoplasms, but which has no risk of bleeding or malignant transformation [12]. To evalute more specifically for FNH, Eovist, a hepatobiliary imaging agent (gadoxetate disodium, manufactured by Bayer, Leverkusen, Germany), was injected with repeat MRI and T1-weighted images were obtained 20 minutes after injection. Unfortunately, there was variable Eovist uptake in the liver lesions with poor uptake in the centrally-located lesion shown in Figure 4, making the diagnosis of FNH less likely. Therefore, since the diagnosis could not be confirmed without conclusive histology, an U/S guided fine needle aspiration (FNA) was performed, which showed only chronic inflammation. An U/S guided core biopsy was then performed which demonstrated significant macrovesicular steatosis, ductular reaction, and somewhat prominent arteries, as well as focally expanded regions of glutamine synthetase immunohistochemical staining in some small fragments, all of which raised the possibility of FNH; however, the sample did not allow for definitive diagnosis. The presence of extensive steatosis, which is rare in FNH, may have altered the imaging characteristics of these lesions, only further complicating the diagnostic picture [13, 14]. Additionally, FNH or FNH-like changes can occur adjacent to a primary hepatic or metastatic neoplasm, which may not be sampled on a small core biopsy [14-16]. Therefore, given ongoing diagnostic uncertainty, the patient then underwent an open wedge biopsy of the liver, which was ultimately diagnostic of a 3 cm steatotic FNH (Figure 5). Ultimately, after four different imaging studies and three invasive procedures, a conclusive diagnosis of the lesions was made and no further intervention or management was deemed necessary. The patient is otherwise doing well. Unfortunately, her sibling, (who did not undergo genetic testing and has not been undergoing surveillance screening) was recently diagnosed with osteosarcoma and thus is most likely a carrier of the same TP53 mutation.

Discussion

Surveillance screening for the early detection of cancer in LFS is an evolving recommendation that stems from data that implementation of such a protocol improves overall survival in this high-risk patient population. However, as more patients undergo these surveillance protocols, providers gain more experience in anticipating and managing the false negatives and false positives that come with additional testing. Our first CPC patient began phlebotomy due to an elevated AFP, which was a false positive from a malignancy standpoint but a true positive for an otherwise incidental finding of hepatic iron overload. Though it ultimately was in the best interest of the patient to treat this finding, it was not an expected consequence of the therapy he received and may not have been identified otherwise. Our second CPC patient is another example of a false positive in which atypical imaging findings required a tissue diagnosis to exclude malignancy. However, this case also highlights the psychosocial challenges associated with screening which include patient and family burden as well as financial and medical difficulties related to additional interventions required to workup a false positive finding [8, 17]. Of note, just as the recommendation for surveillance is an evolving one, so are the techniques used to complete surveillance itself. Current surveillance protocols for early tumor detection typically combine multiple modalities including laboratory studies and imaging. However, the use of circulating tumor deoxyribonucleic acid (ctDNA), which has been studied as a biomarker for response to therapy or a sign of relapse, is also now being studied in pre-clinical models in the context of cancer surveillance for high risk populations (Sangeetha Paramathas, Nathan Lewis, Tanya Guha, Zainab Motala, David Malkin. Assessing the utility of circulating tumor DNA as a surveillance tool for sarcomas and Li-Fraumeni syndrome using a pre-clinical model [abstract]. In: Proceedings of the American Association for Cancer Research Annual Meeting 2017; 2017 Apr 1-5; Washington, DC. Philadelphia (PA): AACR; Cancer Res 2017;77(13 Suppl):Abstract nr 2741. doi:10.1158/1538-7445.AM2017-2741). This is a promising method by which to potentially limit false positives, which are inherent with any screening protocol.

O’Neill et al. demonstrated that whole body MRI, as proposed by the Sick Kids Protocol, is feasible for cancer screening in the pediatric setting and advantageous given the ability to rapidly image multiple body parts and avoid ionizing radiation, but they reported a high rate of incidental findings which required follow-up imaging studies, and in one case, biopsy of a lesion that proved to be benign [18]. In addition, “scanxiety” - the anxiety associated with imaging which is common in solid tumor oncology patients who are waiting to hear whether their tumor is responding to treatment or not  – is equally prevalent in patients receiving surveillance screening [19]. That said, surveillance screening can provide patients with a feeling of empowerment, which can be helpful, particularly in the setting of LFS, a condition in which patients may feel a loss of control and a lack of hope. This is supported by research that demonstrates that parents whose children undergo newborn screening report overall lower stress than parents of children whose diagnosis was made upon clinical presentation [20]. However, it has been shown that there is an increased incidence of hospitalization for children with false positives as compared to those with normal results and that mothers of these children have higher levels of stress as quantified on a parental stress index test [20]. It is known that the most stressful and anxiety-provoking time is between the positive screening test and the confirmatory test and that the psychosocial impact of a false positive can persist even after a negative confirmatory test [21]. Furthermore, many individuals who underwent genetic testing for LFS, irrespective of their carrier status, tended to have more levels of distress [22]. Thus, though the potential medical benefits of early screening are clear, it can come with significant adverse effects on patient and family emotional well-being.

## Conclusions

Surveillance with biochemical and radiographic modalities in patients with known genetic predisposition can decrease the known associated morbidity and mortality of these syndromes. General surveillance appears to be warranted in LFS and results in improved overall survival with the early detection of asymptomatic tumors and early treatment. As providers more uniformly implement such screening protocols, however, it is important to recognize the burdens of such a system and prepare families for the potential adverse psychosocial and medical consequences with regards to false positive and incidental findings. By continuing to study new biomarkers for surveillance, such as ctDNA, and by increasing awareness and communication of specific false positive findings seen in patients undergoing surveillance screening, providers can have a deeper understanding of the burdens and consequences of such protocols. Lastly, as cancer genomics continues to rapidly expand, more genes will likely be identified that explain cancer predisposition syndromes; thus, preventative cancer screening will likely be incorporated more frequently for early cancer detection in numerous other settings.
